# Novel Antihypertensive Peptides Derived from Chicken Foot Proteins

**DOI:** 10.1002/mnfr.201801176

**Published:** 2019-04-10

**Authors:** Francisca I. Bravo, Anna Mas‐Capdevila, Maria Margalef, Anna Arola‐Arnal, Begoña Muguerza

**Affiliations:** ^1^ Universitat Rovira i Virgili Department of Biochemistry and Biotechnology Nutrigenomics Research Group C/ Marcel.lí Domingo n^o^ 1 Tarragona 43007 Spain; ^2^ EURECAT‐Technology Centre of Catalonia Technological Unit of Nutrition and Health Avda. Universitat n^o^ 1 Reus 43204 Spain

**Keywords:** ACE inhibitory peptides, hypertension, mass spectrometry, protein hydrolysates, spontaneously hypertensive rats

## Abstract

**Scope:**

Chicken foot proteins have recently been demonstrated by the group to be a great source of hydrolysates with antihypertensive properties. The aim of this study was to isolate and identify angiotensin I‐converting enzyme inhibitory (ACEI) peptides from chicken foot hydrolysate Hpp11 and to test their antihypertensive properties.

**Methods and Results:**

Peptides are separated into fractions according to their molecular size and hydrophobicity by ultrafiltration and RP‐HPLC, respectively. Subsequent peptide identification in the two fractions that present the highest ACEI activities is carried out by HPLC‐MS. Ten of the identified peptides are synthesized and five of them show ACEI (IC_50_) values lower than 100 µm. The antihypertensive effects of these ACEI peptides after oral administration is evaluated in spontaneously hypertensive rats. The peptides AVFQHNCQE and QVGPLIGRYCG exhibit antihypertensive activity when administered at an oral dose of 10 mg kg^−1^ body weight. The maximal decrease in systolic blood pressure is recorded 6 h after their administration (−25.07 ± 4.21 and −10.94 ± 1.96 mmHg, respectively).

**Conclusion:**

These results suggest that AVFQHNCQE and QVGPLIGRYCG could be used as functional ingredients with antihypertensive effects, although it would be necessary to perform bioavailability and clinical studies to demonstrate their efficiency in humans.

## 1 Introduction

In recent years, hypertension has become a public health concern, being one of the independent risk factor for developing cardiovascular diseases (CVD), the leading global cause of death.^1^ It is well known that a reduction in blood pressure (BP) is beneficial to prevent CVD. In fact, a reduction of 10/5 mmHg in systolic blood pressure (SBP)/diastolic blood pressure (DBP) significantly reduces the relative risk of all major cardiovascular outcomes.^2^ One of the main regulatory systems of the BP is the renin‐angiotensin‐aldosterone system being angiotensin I‐converting enzyme (ACE, EC 3.4.15.1) the key enzyme in this system. ACE belongs to a family of zinc metallopeptidases and produce the cleavage of the C‐terminal dipeptide from angiotensin I to release angiotensin II, a potent vasoconstrictor.^3^ The inhibition of ACE is considered important target for treatment of hypertension.^4^ However, the administration of synthetic ACE inhibitors, such as captopril, lisinopril, and enalapril, has been reported to have adverse side effects.^5^ Thus, there has been an increasing interest in development of the natural antihypertensive compounds and ACE inhibitors as alternative for lowering BP.^6^

In this sense, bioactive peptides from dietary proteins have reported a wide range of biological activities,^7^, ^8^ including antihypertensive effect.^9^, ^10^, ^11^ These peptides are enclosed in the native structure of the proteins and a treatment is needed to release these bioactive sequences.^12^ However, other factors such as the possible structural changes performed during protein digestion and the suitability to be absorbed in the gastrointestinal tract could interfere in the peptides final bioactivity in vivo.^13^ Therefore, although bioactive peptides presenting in vitro ACE inhibitory (ACEI) activity has been extensively reported,^14^ the evidence for their beneficial antihypertensive effects has to be based on the effect after their administration in animal experiments.^15^

Different protein sources have been demonstrated to release ACEI peptides with BP lowering properties after their oral administration,^9^, ^11^, ^16^ including proteins from chicken by products.^17^ These chicken by products are disposed of as waste, producing environmental and economic problems.^18^, ^19^ Therefore, their reuse for the obtainment of bioactive peptides including antihypertensive peptides has received great attention.^17^ In this sense, chicken feet, by products of the poultry industry, have recently been established by our group as an excellent protein source to obtain hydrolysates with antihypertensive properties.^20^ In fact, the BP lowering effect after short and long‐term administration of a low dose of the chicken foot hydrolysate Hpp11 have been recently demonstrated in spontaneously hypertensive rats (SHR) and cafeteria‐diet fed rats.^21^, ^22^ However, the chicken foot bioactive peptides have not been characterized yet. Therefore, the aim of the present study was to isolate and identify the ACEI peptides present in Hpp11. The isolation of the peptides was performed by ultrafiltration followed by two chromatographic steps and their amino acid sequence were identified by mass spectrometry. The antihypertensive effect of peptides that exhibited notable ACEI activity was posteriorly proved in SHR.

## 2 Experimental Section

### 2.1 Chemicals and Reagents

Chicken feet (*Gallus gallus domesticus*) were provided by a local farm (Granja Gaià, La Riera de Gaià, Spain). Protamex solutions (EC 3.4.21.62 and 3.4.24.28, 1.5 AU g^−1^ from *Bacillus licheniformis and Bacillus amyloliquefaciens*) were kindly provided by Novozymes (Bagsværd, Denmark). ACE (angiotensin I‐converting enzyme, EC 3.4.15.1) was purchased from Sigma‐Aldrich (Madrid, Spain), captopril (PubChem CID: 44093) was purchased from Santa Cruz Biotechnology (Dallas, TX, USA) and *o*‐aminobenzoylglicil‐*p*‐nitrofenilalanilprolina (o‐Abz‐Gly‐p‐Phe(NO_2_)‐Pro‐OH) was provided by Bachem Feinchemikalien (Bubendorf, Switzerland). The synthesized peptides (LSETVV, LSGPVKF, AVKILP, VRWEPAPGPV, VGKPGARAPMY, QVGPLIGRYCG, LGIHPDWQFV, and AVFQHNCQE, purity grade ≥ 90 %) were purchased from Caslo Laboratory ApS (Kongens Lyngby, Denmark). Acetonitrile and trifluoroacetic acid were purchased from Sigma‐Aldrich. All other chemical solvents used were of analytical grade.

### 2.2 Preparation of Chicken Foot Hydrolysate

The chicken foot protein hydrolysate Hpp11 was elaborated using the commercial enzymatic solution protamex as previously was described.^20^ Briefly, chicken feet were cleaned, crushed, lyophilized, milled, and sieved using a 2‐mm pore size sieve to obtain a fine chicken foot powder. Chicken foot powder (20 mg mL^−1^, w/v) was resuspended in distilled water and incubated for 90 min in a water bath set at 100 °C at 100 rpm. Subsequently, the protamex enzymatic solution was added at a final concentration of 2.67 µg mL^−1^ (enzyme/substrate ratio, 0.4 AU g^−1^ protein). Hydrolysis was carried out at 50 °C for 2 h at pH 7.0 in a MaxQ Orbital Shaker Thermo Scientific (Thermo Fisher Scientific, Waltham, MA, USA). At the end of the reaction, the enzyme was heat‐inactivated (80 °C, 10 min) in a water bath. Then, the hydrolysate was centrifuged at 10 000 × *g* for 20 min at 4 °C and the supernatant was filtered through a 0.45‐µm membrane; finally, the filtrate was collected for analysis.

### 2.3 Isolation and Identification of ACEI Peptides from Chicken Foot Hydrolysate

### 2.3.1 Step I: Protein Separation Based on Protein Molecular Weight

Hpp11 was subjected to ultrafiltration through two hydrophilic membranes with cut‐off values of 3 and 10 kDa cut‐off (Centripep, Amicon, Inc., Beverly, MA, USA). The obtained fractions (<3 kDa, 3–10 kDa and >10 kDa) were freeze‐dried and kept at −20 °C until use. The protein concentration and ACEI activity were tested for each fraction at least in duplicate and used for fraction selection.

### 2.3.2 Step II: Peptide Separation Based on Peptide Hydrophobicity

Semipreparative reverse phase high performance liquid chromatography (RP‐HPLC) separations of the <3 kDa fraction were performed on an Agilent Series 1260 HPLC equipped with the Agilent OpenLab CDS ChemStation Edition for LC & LC/MS systems A.01.04 software for data acquisition (Agilent Technologies, Santa Clara, CA, USA). A Europa peptide C18 column (25 × 1.0 cm i.d., 5 µm particle size, 120 A˚ pore size) (Teknokroma) was used. Mobile phase A was a mixture of water‐trifluoroacetic acid (1000:1) and mobile phase B contained a mixture of acetonitrile‐trifluoroacetic acid (1000:0.8). Elution was performed in gradient mode as follows: initial conditions 0 % B; 0–40 % B, 0–50 min; 40–45 % B, 50–51 min; 45–90 % B, 51–56 min; and 90–0 % B, 56–57 min. A 10 min post‐run was required for column re‐equilibration. The flow rate was set at 4 mL min^−1^ and analyses were performed at room temperature (RT). Absorbance of the eluent was monitored at 214 nm. The sample concentration was 10 mg mL^−1^ (dissolved in water) and the injection volume was 750 µL. Fractions from the HPLC system were freeze‐dried and kept at −20 °C until use. The protein concentration and ACEI activity were also tested for each fraction at least in duplicate and used for fraction selection.

The two fractions showing the most potent ACEI activity, F.3 and F.6, were subsequently subjected to a second semipreparative RP‐HPLC separation. The chromatographic conditions were similar but the elution was performed with a linear gradient of solvent B in A, going from 10 % to 20 % B over 40 min at RT or from 20% to 30 % B over 40 min for fractions F.3 and F.6, respectively. A flow rate of 4 mL min^−1^ was used in both cases. The sample concentration was 10 mg mL^−1^ (dissolved in water) and the injection volume was 750 µL. The ACEI activity and protein concentration were determined in all subfractions at least in duplicate and used for subfraction selection.

### 2.3.3 Step III: Peptide Identification by HPLC‐MS

For peptide identification, the most active hydrolysate subfractions (F3.3 and F6.6) obtained in the second RP‐HPLC separation were diluted in 0.1 % TFA to a 0.1 mg mL^−1^ final concentration. A 10 µL aliquot of each sample was injected into the linear trap quadrupole (LTQ) Orbitrap Velos‐PRO (Thermo Scientific). Peptides were loaded onto an EASY‐Column (2 cm, ID 100 µm, 5 µm, C18‐A1 precolumn) (Thermo Scientific) and then eluted onto an EASY‐Column (10 cm, ID 75 µm, 3 µm, C18‐A2 analytical column) (Thermo Scientific) at a flow rate of 400 nL min^−1^ on a nanoEasy high‐performance liquid chromatography (HPLC) instrument (Proxeon) coupled to a nanoelectrospray ion source (Proxeon). The mobile phases used consisted of 0.1 % formic acid/2 % ACN (solvent A) and 0.1 % formic acid in 100 % ACN (solvent B). For the F3.3 fraction, the gradient was 0–45 % B over 80 min, from 45–100 % over 20 min and then 10 min at 100 % B. In contrast, for the F6.6 fraction, the gradient was from 0–35 % B over 40 min, from 35–100 % over 10 min and then 10 min at 100 % B. All mass spectra were acquired in positive‐ion mode. Full‐scan MS spectra were (*m*/*z* 50–2000) were acquired with a target value of 1 000 000 at a resolution of 30 000 at 400 *m*/*z*, and the 15 most intense ions were selected for collision‐induced dissociation fragmentation in the LTQ with a target value of 10 000 and a normalized collision energy of 35 %. Precursor ion charge‐state screening and monoisotopic precursor selection were enabled. Singly charged ions and unassigned charge states were rejected. Dynamic exclusion was enabled with a repeat count of 1 and an exclusion duration of 30 s.

Proteome Discoverer 1.4.288 (Thermo) with MASCOT 2.4.1.0 was used to search the IPI_chicken_3.81 fasta database (25992 sequences). The following database search parameters were used: peptide tolerance, 10 ppm; fragment ion tolerance, 0.8 Da; no enzyme and variable modification, methionine oxidation. During peptide identification, probability scores greater than the score fixed by Mascot were considered as significant with a *p*‐value minor than 0.05. The automatic decoy database search function in Protein Discover was enabled to allow estimation of the false discovery rate (FDR).

The identified sequences were subsequently chemically synthesized by Caslo Laboratory ApS.

### 2.4 ACEI Activity

ACEI activity was measured according to Mas‐Capdevila et al.^21^ The fluorescence measurements were performed after 30 min in a multiscan microplate fluorimeter (FLUOstar optima, BMG Labtech, Offeuburg, Germany). The excitation and emission wavelengths were 360 and 400 nm, respectively. The software used to process the data was FLUOstar control (version 1.32 R2, BMG Labtech).

A nonlinear fit was performed on the experimental data to calculate the 50 % inhibitory concentration values (IC_50_) with the PRISM version 4.02 program for Windows (GraphPad Software, Inc. San Diego, CA, USA). ACEI activity was expressed as a percentage (%) or IC_50_ (µg µL^−1^ solution). The determination of the ACEI activity of the samples was performed at least in duplicate. Data are represented as a mean value ± standard deviation (SD).

Protein content was determined by the bicinchoninic acid method using the standard Pierce BCA Protein Assay (ThermoFisher Scientific). The assay was conducted according to the manufacturer's instructions in a microplate format. A calibration standard curve was prepared with seroalbumin bovine. Determination of the protein content was performed at least in duplicate. The results are expressed as the mean ± SD.

### 2.5 Measurement of Blood Pressure

Male SHR (17–20 weeks old, weighing 350–400 g) were obtained from Charles River Laboratories España S.A. (Barcelona, Spain). The animals were singly housed in animal quarters at 22 °C with a 12 h light–dark period. They were fed with a standard diet based on chow Panlab A04 (Panlab, Barcelona, Spain) and had access to tap water *ad libitum*.

The SBP and DBP in the animals were measured using the tail‐cuff method 23 with a noninvasive BP system model (Letica, Hospitalet, Barcelona, Spain). The rats were given a single dose of 10 mg kg^−1^ body weight (bw) of the synthesized peptides, including VGKPGARAPMY, QVGPLIGRYCG, AVFQHNCQE, LSGPVKF, or AVKILP, dissolved in tap water in a total volume of 1.5 mL. Peptides were administered to the SHR by gastric incubation through an acute administration between 9 and 10 in the morning.

Positive control rats received 50 mg kg^−1^ bw of captopril, a known ACE‐inhibitor, also dissolved in 1.5 mL of tap water, and the negative control rats received 1.5 mL of tap water. SBP was measured in the rats before peptide administration as well as 2, 4, 6, 8, and 24 h post‐administration. Before the measurement, the animals were kept at 38 °C for 10 min to detect the pulsations of the tail artery. The changes in SBP and DBP were expressed as the differences in these variables before and after the administration of the different peptides. Data are shown as the mean values ± standard error of the mean (SEM) for a minimum of six experiments. Before starting the experiment, all animals were accustomed to the process with a 2‐week training period.

The animal protocol followed in this study was approved by Spanish Royal Decree 223/1988 and by the Bioethical Committee of Universitat Rovira i Virgili (reference number 8868 by Generalitat de Catalunya).

Differences between treatments were analyzed by two‐way analysis of variance (two‐way ANOVA). All the analyses were performed using IBM SPSS Statistics (SPSS, Chicago, IL, US). Outliers were determined using Grubbs’ test. Differences between groups were considered significant when *p* < 0.05.

## 3 Results

### 3.1 Identification of ACEI Peptides

**Figure 1:**
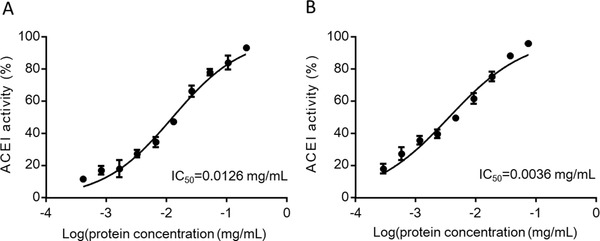
Determination of the angiotensin I‐converting enzyme inhibitory (ACEI) activity expressed as the IC_50_ for A) Hpp11 hydrolysate and B) the Hpp11 hydrolysate <3 kDa fraction. The IC_50_ values were determined by curve fitting with a nonlinear regression analysis. The experimental data in each graphic correspond to two different assays in duplicate.

Hpp11 was first subjected to ultrafiltration through 3 kDa and 10 kDa cut‐off membranes to separate the peptides according to their molecular size. ACEI activity was determined in the following three obtained fractions: <3 kDa, 3–10 kDa and >10 kDa. The percentages of inhibition obtained were 95.98 ± 0.72 for the <3 kDa fraction, 13.25 ± 2.86 for the 3–10 kDa fraction and 30.50 ± 2.24 for the >10 kDa fraction at a protein concentration of 74.2, 78.3, and 41.7 µg mL^−1^, respectively. The ACEI activity of the <3 kDa fraction was the most active and the expressed as IC_50_ was three and a half times higher than activity found for Hpp11 hydrolysate (3.6 µg vs 12.6 µg mL^−1^) (**Figure** 1). These results indicate that the ACEI activity of Hpp11 is mainly due to small peptides.

**Figure 2:**
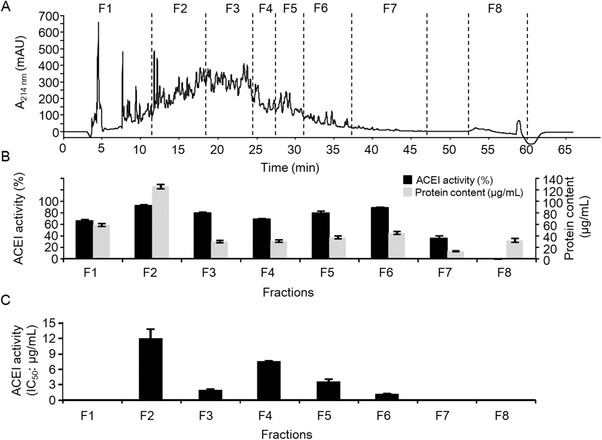
A) Fractionation by RP‐HPLC at a semipreparative scale for the <3 kDa fraction obtained from Hpp11. The collected fractions were termed with F followed by a number (F1–F8). The angiotensin I‐converting enzyme inhibitory activity expressed as B) the percentage of inhibition and C) IC_50_ (µg mL^−1^) of the collected fractions from the semipreparative RP‐HPLC system. The data are expressed as the mean ± standard deviation for a minimum of two measurements. The protein contents of the fractions were estimated using the bicinchoninic acid assay.

**Figure 3:**
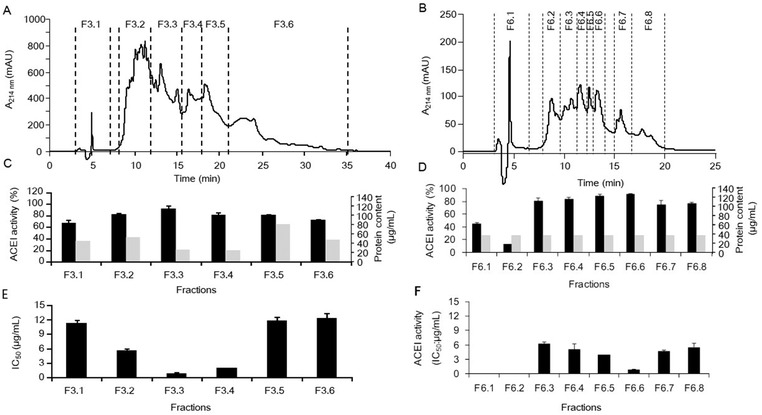
Fractionation by RP‐HPLC at a semipreparative scale for fractions A) F3 and B) F6 obtained from the first RP‐HPLC separation. The collected subfractions are termed with the name of the original fraction followed by a point and a number. The angiotensin I‐converting enzyme inhibitory activity expressed as C,D) the percentage of inhibition and E,F) IC_50_ (µg mL^−1^) of the subfractions from the second separation of F3 and F6, respectively. The data are expressed as the mean ± standard deviation for a minimum of two measurements. The protein contents of the fractions were estimated using the bicinchoninic acid assay.

The <3 kDa fraction was subjected to semipreparative RP‐HPLC to separate the peptides according to their peptide hydrophobicity and eluted as described in Section 2.3.2. The base peak chromatogram for this fraction can be seen in **Figure** 2A, showing that the hydrolysate was a complex mixture of peptides. Thus, it was divided into eight fractions (named from F1 to F8), which were collected and lyophilized, and their ACEI activities were measured (Figure 2A,B). Fractions F2 to F6 showed high ACEI activity (≥70 %). The ACEI activity of these selected fractions was also determined and expressed as IC_50_ values (µg of protein per mL) to identify the fraction with the largest amounts of bioactive peptides. Most of the ACEI activity from the <3 kDa fraction occurred in fractions 3 and 6 (F3 and F6), which showed IC_50_ ≤ 2 µg mL^−1^ (1.99 and 1.24 µg mL^−1^, respectively). These two fractions were further purified by a second RP‐HPLC step. **Figure** 3A,B shows the RP‐HPLC pattern obtained from F3 and F6. Fraction F3 was subdivided into six new subfractions (from F3.1 to F3.6) and F6 was subdivided into eight new subfractions (from F6.1 to F6.8). All fractions showed a specific ACEI activity ≤12 µg mL^−1^ (IC_50_) (Figure 3B–E). However, fractions F3.3 and F6.6 were remarkably the most active, with IC_50_ values of 0.83 and 0.86 µg mL^−1^, respectively (Figure 3D,E).

**Figure 4:**
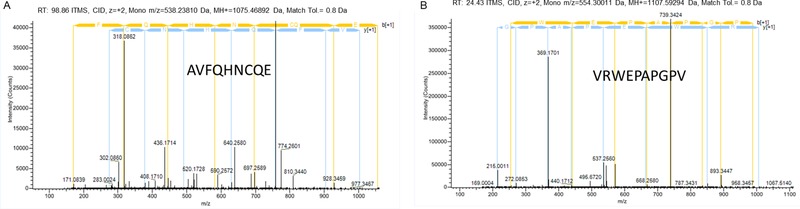
MS/MS spectrum of the doubled charged ions *m*/*z* A) 538.2 and B) 554.3. Following sequence interpretation and database searching, the peptides were identified as AVFQHNCQE and VRWEPAPGPV, respectively. The MS/MS spectra were acquired with linear trap quadrupole‐Orbitrap mass spectrometry. The sequences of these peptides are displayed with the fragment ions observed in the spectra.

The analysis of these subfractions by mass spectrometry allowed the identification of 772 peptides in fraction F3.3 and 248 peptides in fraction F6.6. Eight of these peptides were subsequently selected and synthesized. Peptide selection was performed according to their high sign intensity, peptide length, and amino acid sequence (**Table** 1). As an example, **Figure** 4A,B shows the MS/MS spectrum of ion *m*/*z* 538.2 and 554.3 corresponding to the sequences AVFQHNCQE and VRWEPAPGPV, respectively. The synthesized peptides showed a wide range of ACEI activity percentages from 0% to 92% (**Table** 2). From the eight peptides analyzed, only five peptides showed an ACEI activity higher than 50 %. Their IC_50_ values were lower than 100 µm. Two of these peptides stood out for their high ACEI activity, sequences AVKILP and QVGPLIGRYCG, which showed IC_50_ values as low as 7.06 and 11.01 µm, respectively.

**Table 1:** Identification of the peptides contained in the obtained RP‐HPLC subfractions showing the highest angiotensin I‐converting enzyme inhibitory activities

Fraction	Sequence^a^	Theoretical M.W.	MH+ [Da]	*m*/*z* [Da]	Charge
F3.3	VGKPGARAPmY^b^	1146.4	1162.59	581.80	2
F3.3	QVGPLIGRYCG	1162.4	1162.61	581.80	2
F3.3	LGIHPDWQFV	1211.4	1211.62	404.54	3
F3.3	AVFQHNCQE	1075.2	1075.47	538.24	2
F6.6	LSETVV	646.7	647.36	324.18	2
F6.6	LSGPVKF	746.9	747.44	374.22	2
F6.6	AVKILP	639.8	640.44	320.72	2
F6.6	VRWEPAPGPV	1107.3	1107.59	554.30	2

a Amino acids are designated using their one letter codes

b m = Oxidation of methionine

**Table 2:** The angiotensin I‐converting enzyme inhibitory activities of the synthetic peptides expressed as percentages and IC_50_ values

Fraction	Sequence	Peptide concentration [µm]	ACEI activity	
			**%** a	IC_50_ b [µm]
F3.3	VGKPGARAPmY	174.5	75.8 ± 0.06	29.7
F3.3	QVGPLIGRYCG	161.4	87.2 ± 0.33	11.0
F3.3	LGIHPDWQFV	137.6	0.0 ± 0.0	>137.6
F3.3	AVFQHNCQE	142.8	90.4 ± 0.02	44.8
F6.6	LSETVV	128.9	12.9 ± 0.71	>515.4
F6.6	LSGPVKF	122.7	58.4 ± 4.27	80.9
F6.6	AVKILP	101.4	91.8 ± 0.03	7.1
F6.6	VRWEPAPGPV	150.5	21.7 ± 2.30	>150.0

a Percentage of ACEI activity showed at the indicated protein concentration.

b Concentration of peptide needed to inhibit 50 % of the original ACE activity.

### 3.2 Antihypertensive Activity of the Synthetic Peptides

**Figure 5:**
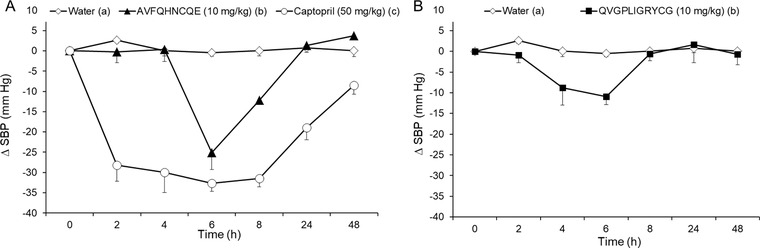
Decrease in the systolic blood pressure (SBP) in spontaneously hypertensive rats after the administration of A) water, captopril (50 mg kg^−1^ bw), or AVFQHNCQE (10 mg kg^−1^ bw) or B) after the administration of water and QVGPLIGRYCG (10 mg kg^−1^ bw). The data are expressed as the mean ± SEM. All of the experimental groups include a minimum of six animals. Different letters represent significant differences (*p* < 0.05). *p* was estimated by two‐way ANOVA.

The antihypertensive effects of the peptides showing the lowest IC_50_ values were evaluated in SHR (**Figure** 5). Prior to oral administration of the different peptides, SHR presented SBP values of 208.5 ± 8.0 mmHg and DBP values of 163.3 ± 4.6 mmHg (*n* = 6 for each treatment). The SBP and DBP of SHR administered water did not significantly change during the 48 h duration of the experiment. As expected, the administration of captopril (50 mg kg^−1^ bw) resulted in an important decrease in the SBP of SHR from 2 h after its administration, with the maximum decrease (−33.67 ± 1.94 mmHg) observed at 6 h post‐administration. With respect to DBP, captopril administration also produced a significant reduction, reaching a maximum decrease 4 h post‐administration (−32.97 ± 8.80 mmHg). The antihypertensive effect was maintained to 48 h after captopril administration.

From the five peptide sequences administered to SHR, including VGKPGARAPMY, QVGPLIGRYCG, AVFQHNCQE, LSGPVKF, and AVKILP, the peptides AVFQHNCQE and QVGPLIGRYCG exerted antihypertensive effects when administered at 10 mg kg^−1^ bw to SHR (Figure 5A,B). Nevertheless, AVFQHNCQE was the most active peptide. The administration of this peptide produced a significant decrease in both the SBP and DBP in SHR (−25.07 ± 4.21 mmHg and −17.65 ± 3.24 mm Hg, respectively). The maximum decrease in SBP caused by AVFQHNCQE was observed 6 h post‐administration while the maximum decrease in DBP was registered at 2 h. BP returned to initial values 24 h after administration. Regarding QVGPLIGRYCG, it was also observed that this peptide produced a significant decrease in SBP (−10.94 ± 1.96 mm Hg) 6 h post‐administration. Nevertheless, no significant reduction in DBP was observed when this peptide was administered. The BP of SHR after VGKPGARAPMY, LSGPVKF, and AVKILP administration did not change during the experiment (data not shown).

## 4 Discussion

It is well‐known that the hydrolysis of dietary proteins generates a large number of peptides, including some with potentially bioactive activities.^24^ In this sense, the hydrolysis of chicken protein from byproducts has been reported to be a good source for obtaining ACEI and antihypertensive peptides.^17^ In a previous study performed by our group, chicken feet dissolved in water were heated at 100 °C and hydrolyzed using protamex to obtain the hydrolysate Hpp11, which was able to reduce BP when administered to SHR at low doses.^20^ The bioactivity of this hydrolysate was assumed to be due to the presence of specific peptide sequences in Hpp11. Thus, the aim of this study was to identify the antihypertensive peptides present in the hydrolysate Hpp11. To assess this objective, Hpp11 was subjected to ultrafiltration using hydrophilic membranes with 3 and 10 kDa cut‐off values and ACEI activity of the different obtained fractions was measured. The results showed that the <3 KDa fraction presented the highest ACEI activity. This finding attributes the highest ACEI activity to small peptides present in this hydrolysate. Similar results were reported by other authors for hydrolysates obtained from other protein sources.^25^, ^26^, ^27^ Subsequently, the <3 kDa fraction was subjected to a two‐step RP‐HPLC analysis resulting in obtaining of two subfractions (F3.3 and F6.6) selected according to their high ACEI activity. The subsequent analysis of subfractions F3.3 and F6.6 by mass spectrometry allowed the selection of eight peptides (LSETVV, LSGPVKF, AVKILP, VRWEPAPGPV, VGKPGARAPMY, QVGPLIGRYCG, LGIHPDWQFV, AVFQHNCQE), which were subsequently synthesized. As far as we know, none of these peptides had been previously identified to have any bioactivity (search carried out in the BIOPEP database as of October 2018, http://www.uwm.edu.pl/biochemia/index.php/pl/biopep).

The ACEI activity, expressed as the IC_50_ value, was lower than 100 µm for five of these identified amino acid sequences, indicating their potential roles as ACE inhibitors. This finding is based on the fact that all the selected peptides comprised between 6–11 amino acids; it has been previously reported that peptides presenting high ACEI activity are short in length and comprising 3–12 amino acids.^6^ It has also been previously reported that the amino acid composition of the three consecutive C‐terminal positions plays an important role in ACE competitive inhibition.^28^ However, the amino acid residues required for ACE inhibition at the C‐terminal position can be different depending on the peptide size.^29^ In general, the presence of hydrophobic aromatic amino acids (Tyr, Phe, or Try), or amino acids with hydrophobic branched side chains (Val, Leu, or Ile) lead to an increase in ACE inhibitory activity.^14^ Additionally, many identified ACE inhibitors contain Pro at the C‐terminal end. In fact, Pro has a rigid ring structure that can lock the carboxyl group into a favorable conformation, making it able to interact with positively charged residues in the active site of the enzyme.^30^ Regarding the N‐terminal position, the presence of branched aliphatic amino acids such as Gly, Val, Leu, and Ile has been reported as good ACE substrates.^31^ Thus, the presence of the Pro residue at AVKILP C‐terminus, the Tyr residue at the C‐terminus and the Val residue at the N‐terminus in VGKPGARAPMY, and the Phe residue at the C‐terminus and Leu at the N‐terminus in LSGPVKF may contribute to their ACEI activity. Regarding the peptide AVFQHNCQE, the presence of glutamic acids in its sequence may contribute to its ACEI effect. Indeed, it is known that glutamic acid may cause a net negative charge, and the interaction of negatively charged peptides with ACE could chelate zinc atoms, which is a component of the ACE active center.^32^ However, the peptide sequence for QVGPLIGRYCG, one of the most potent ACE inhibitors identified in the Hpp11, did not have any particular structural features that could be responsible for its activity. Nevertheless, the presence of Tyr at the third position before the final position at the C‐terminus or its conformation in solution could highly favor its ability to inhibit ACE.^30^ In this sense, peptide conformation, that is the structure adopted in the specific environment of the binding site, has been suggested to enhance the inhibitory ability of long‐chain peptides.^33^, ^34^

Additionally, two of the peptides, AVKILP and QVGPLIGRYCG, stood out for their high ACEI activities, showing IC_50_ values as low as 7.06 and 11.01 µm, respectively. Although the ACEI activity of the drug captopril is higher than that presented by these peptides, the interest in dietary bioactive compounds is increasing significantly because they present high tissue affinity, specificity, and efficiency in promoting these health effects.^35^ Moreover, the ACEI values of chicken foot‐derived peptides are similar than those observed for known antihypertensive peptides, such as the fermented milk‐derived peptides IPP, VPP, and LHLPLP, which showed similar IC_50_ values (5, 9, and 5.5 µm, respectively).^25^, ^36^ Other byproduct chicken‐derived peptides have been reported to exert high ACEI activities, but their activities ranged from 34 to 254 µm.^37^, ^38^

Many peptides that present ACEI activities in vitro do not exhibit antihypertensive properties. In fact, after their oral administration, the peptides may be susceptible to degradation by gastrointestinal enzymes and by brush border, blood serum, and/or intracellular peptidases before being transferred into the bloodstream.^14^ Additionally, even though some peptides are resistant to digestive enzymes, large peptides (>6 amino acid residues) may not be absorbed into small intestinal epithelial cells.^13^ Therefore, testing the in vivo antihypertensive effects of the peptides is necessary for validation. To assess their effects in vivo, the ACEI peptides identified in Hpp11 were administered to SHR, an experimental animal model that best mimics essential hypertension in humans.^39^

The VGKPGARAPMY, LSGPVKF, and AVKILP peptides sequences did not exhibit BP‐lowering effects. Interestingly, the AVKILP peptide, which showed the most potent ACEI activity, did not present antihypertensive effects when administered to SHR, suggesting that modifications incurred during gastrointestinal digestion could inactivate this peptide. In contrast, the AVFQHNCQE and QVGPLIGRYCG peptides decreased BP after their oral administration (−25.07 and −10.94 mm Hg of SBP at 6 h post‐administration, respectively), with AVFQHNCQE being the most active peptide. Interestingly, this peptide was also able to reduce DBP, suggesting its huge potential as an antihypertensive agent. Our findings agree with those demonstrating the potential antihypertensive properties of bioactive peptides from chicken by products including chicken bone.^39^ Furthermore, the ACEI peptides IKW, LKP, and IVGRPRHQG, which were isolated from chicken muscle, showed similar antihypertensive effects as AVFQHNCQE but needed to be administered at a higher dose of 60 mg kg^−1^ bw.^40^

## 5 Conclusions

Novel ACEI peptides have been identified in the chicken foot hydrolysate Hpp11. Moreover, the antihypertensive properties of the QVGPLIGRYCG and AVFQHNCQE peptides have been demonstrated in this study for the first time. The potential of these peptides for use in functional foods to mitigate hypertension appears to merit further clinical studies in humans.

## Conflict of Interest

The authors declare no conflicts of interest.
